# Work stress and its association with suicidal ideation, health and presenteeism during the COVID-19 pandemic: cross-sectional study in the UK health and university workforce

**DOI:** 10.1192/bjo.2025.10069

**Published:** 2025-07-21

**Authors:** Christina van der Feltz-Cornelis, Jennifer Sweetman, Dorota Merecz-Kot, Carlota de Miquel, Fidan Turk, Beatriz Olaya

**Affiliations:** Department of Health Sciences, University of York, UK; Hull York Medical School, University of York, UK; Institute of Health Informatics, University College London, UK; Institute of Psychology, University of Lodz, Poland; Epidemiologia dels trastorns mentals i de l’envelliment, Institut de Recerca Sant Joan de Déu (IRSJD), Esplugues de Llobregat, Spain; Centro de Investigación Biomédica en Red de Salud Mental (CIBERSAM), Madrid, Spain; Department of Clinical and Health Psychology, Autonomous University of Barcelona, Spain; Department of Psychology, University of Exeter, UK

**Keywords:** Work stress, suicidal ideation, presenteeism, mental health, physical health

## Abstract

**Background:**

Work stress levels rose among health and educational workforces during the COVID-19 pandemic, and can affect employee well-being and organisational efficiency.

**Aims:**

To explore the association of work stress with mental health, including suicidal ideation and physical health, as well as presenteeism, as aspects of organisational efficiency in UK healthcare and university workers.

**Method:**

A total of 328 UK participants completed self-report questionnaires between April 2022 and September 2023 in the context of the European Platform to Promote Wellbeing and Health in the Workplace (EMPOWER) study. Cross-sectional analyses were conducted.

**Results:**

Two hundred and ninety-two (90%) employees reported work-related stress (Mini-Psychosocial Stressors at Work Scale). Depressive, anxiety and somatic symptoms were reported (61, 55 and 75%, respectively); 11% of the participants reported suicidal ideation (Patient Health Questionnaire 9) and 56% reported presenteeism (iMTA Productivity Cost Questionnaire). Psychological and somatic symptoms were worse when suicidal ideation or presenteeism was reported. Stressful work factors included having too much work to do (63%), a bad working atmosphere (28%), poor work–home balance (32%) and working hours hindering private life (35%). Spearman correlations showed significant associations between work stress and suicidal ideation (0.225), depressive (0.290), anxiety (0.299) and somatic symptoms (0.245) and presenteeism (0.311), but not with having a chronic medical condition.

**Conclusion:**

Given the association between work stress, suicidal ideation and presenteeism, research should explore how psychosocial risk factors linked to work stress could be reduced for healthcare and higher education employees. The findings warrant the development of policies to address work stress, and to provide employee support for suicidal ideation and presenteeism in the work setting.

Work stress occurs frequently and is associated with mental health conditions such as depression^1^ and somatic conditions, including obesity, diabetes and breast cancer, that have been reported to occur more often in the case of night shifts and long working hours, plus cardiovascular conditions, arthritis and respiratory conditions.^2–6^ Moreover, work stress in people with chronic medical conditions can increase mortality risk.^6^ In the UK during the COVID-19 pandemic, self-reported work stress levels across all industries increased, compared with prepandemic, to prevalence rates of 2070 per 100 000 workers (95% CI 1.940 to 2.200) in the period 2020/21–2022/23, and those higher rates were sustained in 2024.^7^ Compared with all industries, the broad industry categories of human health and social work (3530 cases per 100 000 workers, 95% CI 3.100 to 3.950), education (2720 cases per 100 000 workers, 95% CI 2.330 to 3.110) and professional/scientific (2310 cases per 100 000 workers, 95% CI 1.830 to 2.790) had significantly higher average rates.^8^ However, although the emerging research describes high work stress levels during the pandemic among healthcare^9–12^ and university staff^13,14^ in the UK, some aspects merit further exploration.

Work stress-related distress can include suicidal ideation and lead to occupational suicide, with serious individual and societal consequences. A case that shocked France is described in Box 1.

Box 1 Suicide court case following ‘institutional harassment’ in a company in France

In 2019, the CEO, two former executives and four other executives of the company France-Telecom (now Orange) received a verdict that they were to be jailed or given suspended sentences in a court case over a restructuring policy linked to suicides among employees in the 2000s. It happened during a major restructuring of the company that affected thousands of employees. At the time, the newly privatised company was in the throes of a major reorganisation. The CEO was trying to cut 22 000 jobs and retrain at least 10 000 workers. The executive team and managers created an atmosphere of fear by transferring some employees away from their families, leaving them behind when offices were moved or assigning them demeaning jobs. France Telecom employees took their own lives between 2008 and 2009 and the CEO stepped down as a result of the deaths. The French court spoke of institutional harassment.^15^

Occupational suicide illustrates the importance of societal surveillance of work stress in companies; however, scientific research into work stress-related suicide or suicidal ideation in employees to date is limited. A recent study analysing prepandemic survey data in the French national working population indicated that several psychosocial work factors were associated with suicidal ideation, such as too many and too difficult tasks, low influence and potential for development, low meaning at work, low sense of community, role conflict, job insecurity, temporary employment, changes at work and violence in the work context.^16^ Other recent studies have reported an association between elevated suicide rates and work stress. Among farm workers in the USA^17^ and in Bangladesh,^18^ suicide rates were related to social isolation, stressful circumstances at work, mental health conditions and somatic illnesses. Among veterinarians in Norway,^19^ suicide rates were related to frequent involvement in euthanising animals. In Italy, gender, job security, level of education and number of hours worked were associated with increased suicide risk in agricultural, fishery, forestry and hunting workers.^20^ Research with employees of general hospitals in the USA has reported suicide rates related to work stress during the COVID-19 pandemic.^21^ To date, this topic has not been extensively examined in the UK context.

In addition to occupational suicide, both mental health and work stress have been associated with presenteeism, defined as attending work but not fully functioning due to health problems,^22–24^ problems doing their work or studying because of psychological or physical symptoms^13^ or, in Medical Technology Assessments, reduced productivity while at paid work.^25^ Presenteeism has consequences for both individuals and employers;^26,27^ it has been considered ‘the biggest threat to workplace productivity in the UK and is characterised by tired, unmotivated and unwell employees who attend work regardless of how bad they’re feeling’.^28^ The cost of poor mental health to UK employers was estimated at £53–56 billion in 2020–2021, of which £24.8–27.6 billion was attributed to presenteeism. This cost actually may be higher, because not only does it cause productivity to drop, costing employers money, ‘but it also adversely impacts workplace morale, health and safety and the wellbeing of employees’.^29^

## Rationale

Following the recommendation of a report that found high levels of mental health conditions in employees, often unbeknownst to the employer,^30^ in 2018 the Health and Safety Executive gave employers the legal duty to protect employees from stress at work by performing a risk assessment and acting on it.^31^ This can support employers in identifying employees with high stress levels and in providing them with resources to address their work stress, such as identifying risk factors at the workplace, which is imperative given the potential link to adverse outcomes such as suicide.^16,20^ Because work stress levels rose especially in UK health and educational workforces during the COVID-19 pandemic, this study aims to explore the association of work stress with suicidal ideation and mental and physical health, as well as presenteeism as an aspect of organisational efficiency in those workforces.

The research focusing on occupational suicide risks underlines the role of occupational stressors.^32^ According to an analysis of occupational risks factors for suicide in England,^33^ there are three groups of factors related to occupational suicide:

- (a) work characteristics, such as work stress, low pay and long hours;
- (b) people characteristics, such as a previous history or risk of mental illness that can be related to the choice of a specific occupation;
- (c) occupation-related ready access to knowledge about lethal suicide methods and the means to put them to use, such as in healthcare personnel.

Of course, classic individual factors such as health status and a history of suicidal behaviour in the family of origin additionally increase the occupational risk of suicide. This is the reason we decided to examine the co-occurrence of professional (stress, presenteeism, absenteeism) and non-professional factors (health status) in their relationship with suicidal behaviour.

## Objectives of the study reported in this paper

- To report levels of work stress, suicidal ideation, symptoms of depression and anxiety and the burden of somatic symptoms, chronic medical conditions, job roles and presenteeism experienced by employees of a university and National Health Service (NHS) Trusts in the UK.
- To explore the association between the aforementioned phenomena.
- To explore how any groups that might be identified on the basis of participants’ levels of (a) presenteeism and (b) suicidal ideation differ from each other in terms of health status and levels of occupational stress.

## Hypotheses

We hypothesise that work stress, suicidal ideation, mental health conditions, somatic symptom burden, chronic medical conditions, patient-facing job roles and presenteeism are prevalent and positively interrelated in healthcare and university workers in the UK.

## Method

### Study design

This paper reports a cross-sectional analysis of baseline data, which were collected in the context of the European Platform to Promote Wellbeing and Health in the Workplace (EMPOWER) study in the UK. The methodology of this study is described extensively elsewhere^34^ and is summarised below for this study. We follow the STROBE guidelines for reporting observational studies;^35^ a checklist is included in Supplementary Table 1 (available at https://doi.org/10.1192/bjo.2025.10069).

### Setting

We recruited employees from public agencies in the UK, namely services of four National Health Service Trusts and six departments of one university. Participants completed their self-report assessments using online tools, which required that all participants have an internet-connected telephone.^34^ The recruitment period was chosen to allow recruitment of at least 218 employees. In order to reach the expected number of participants in the control trial, we chose an initial recruitment period of 12 months,^34^ which was finally extended to 18 months (from 25 February 2022 to 30 September 2023). An overview of COVID-related restrictions for NHS employees in that period is listed in the Supplementary material.

### Participants

All employees from participating NHS Trust localities and from participating university departments were invited by email to participate. The inclusion criteria for participants were:

- (a) being aged 18 years or older;
- (b) having a mobile telephone with internet access;
- (c) having sufficient knowledge of the local language;
- (d) giving informed consent.

Participants were invited to download a digital application (app) with digital informed consent and baseline questionnaires.

### Ethical approval

The authors assert that all procedures contributing to this work comply with the ethical standards of the relevant national and institutional committees on human experimentation, and with the Helsinki Declaration of 1975 as revised in 2013. All procedures involving human participants for this study were approved by NHS HRA and Health and Care Research Wales (reference no. 22/HRA/0629).

### Variables and measurements

Variables and measurements included in the analysis are shown in Table 1.

**Table 1:** Variables and measures used in the analysis

Variable	Questionnaire	Reference	Items	Scoring
Stressors in the social context at work	Mini-Psychosocial Stressors at Work Scale (Mini-PSWS)	Reduced version of Psychosocial Stressors at Work Scale (PSWS)^43^ theoretically based on the European Framework for Psychosocial Risks Management (PRIMA-EF;^44–46^ shown in Supplementary file)^47^	The Mini-PSWS contains 16 items. Each item indicates a stressor and, if checked by the participant, the level of stress for each of the individual stressors was captured using the question, ‘How stressed does this feature make you feel?’. Available response options were scored between 0 and 4, where 0 indicated that participants did not feel stressed at all and 4 that participants felt very stressed in relation to the identified stressor.	Mean and individual item scores from the Mini-PSWS. Ascertain the types of stressors frequently reported, and the associated levels of stress. Summing up the indicated levels of stress per stress factor, and as a total. Total score varies from 0 to 72, with higher scores indicating higher levels of work stress. PSWS level of stress was measured by the question, ‘How stressed does this feature make you feel?’, with response options 0 (not at all) to 4 (very much).
Depressive symptoms	Patient Health Questionnaire (PHQ-9)	PHQ-9 is a brief, reliable and valid instrument that scores each of the DSM-IV criteria for major depressive disorder. It has been extensively validated for measuring distress and depressive symptoms, including severe-level depressive symptoms.^48^	PHQ-9 consists of 9 items exploring depressive symptoms over the past 2 weeks. Each item is scored from 0 (not at all) to 3 (nearly every day).	For the complete PHQ-9, scores of 0–5, 6–10, 11–15 and 16–20 represent mild, moderate, moderately severe and severe depressive symptoms, respectively, where a score of 10 is used as a cut-off indicating that diagnostic and treatment measures may be needed.^29^
Current depressive symptoms	Items 1–8 of PHQ-9 (PHQ8)	These items assess current depression without taking suicidal ideation into account. PHQ-8 is a valid and reliable tool for measurement of depressive symptoms.^40^	The first eight items of PHQ-9. Each item is scored from 0 (not at all) to 3 (nearly every day).	The total score for PHQ-8 can vary from 0 to 24, with higher scores indicating higher levels of depressive symptoms.
Suicidal ideation	Item 9 of PHQ-9	This item specifically assesses suicidal ideation.^48^	The final item from PHQ-9: Over the past 2 weeks, how often have you been bothered by any of the following problems? Thoughts that you would be better off dead, or thoughts of hurting yourself in some way. Response options: 0 not at all, 1 several days, 2 more than every other day, 3 nearly every day.	If a person reports any suicidal ideation on this item, this is scored as suicidal ideation in the analysis (1) versus no suicidal ideation (0).
Anxiety	Generalised Anxiety Disorder Questionnaire (GAD-7)	GAD-7 is a self-report questionnaire that measures symptoms of anxiety during the past 2 weeks. GAD-7 is a reliable and well validated questionnaire. It has been extensively validated for measuring distress and anxiety symptoms, including severe-level anxiety symptoms.^49^	For each item of this 7-item questionnaire, scores range from 0 (not at all) to 3 (nearly every day).	Total scores range from 0 to 21, with higher scores indicating higher levels of anxiety symptoms. Scores of 0–5, 6–10, 11–15 and 16–21 represent mild, moderate, moderately severe and severe anxiety symptoms, respectively, where a score of 10 is used as a cut-off indicating that further diagnostic and treatment measures may be needed.^39^
Somatic symptoms	Patient Health Questionnaire (PHQ-15)	The PHQ-15 somatic scale assesses the severity of physical symptoms. This is a reliable and well-validated instrument. It has been extensively validated for measuring distress-related somatic symptoms, including at the clinical level.^50^	PHQ-15 consists of 15 items. Each item is scored from 0 (not at all) to 3 (nearly every day).	Total score varies from 0 to 45, with higher scores indicating higher levels of depressive symptoms. Scores of 0–5, 6–10, 11–15 and 16+ represent mild, moderate, moderately severe and severe somatic symptoms, respectively, where a score of 10 is used as a cut-off indicating that further diagnosis and treatment may be needed.^39^
Chronic medical conditions	Modified CBS Statline questionnaire	Self-reported chronic conditions diagnosed by a medical professional in the past 12 months, including asthma, chronic bronchitis, chronic obstructive pulmonary disease; serious cardiac condition or heart attack, stroke, high blood pressure; gastric ulcer, serious bowel/intestinal disorder (e.g. Crohn’s disease), gallstones or gallbladder illness; liver disease; kidney stones, serious renal disease; chronic urinary tract infection; diabetes; thyroid disease; disc prolapse, arthrosis or rheumatoid arthritis; epilepsy, Parkinson’s, multiple sclerosis; migraine; cancer.^51^	A single question was asked to ascertain whether participants had experienced any chronic medical conditions over the preceding 12 months that had been diagnosed by a medical professional.	If any conditions were indicated, participants were scored 1 to reflect the presence of a chronic medical condition. If no conditions were indicated, participants were scored 0.
Presenteeism	iMTA Productivity Cost Questionnaire (iPCQ)	Presenteeism was measured using iPCQ question 7.^25^ Although iPCQ is a reliable and well-validated instrument for absenteeism, validating the measure for presenteeism is a challenge due to the lack of a gold standard.^52^	During the last 4 weeks, have there been days in which you worked but during this time were bothered by any kind of problems that made it difficult for you to get as much work finished as you normally do?	Reduced productivity while at paid work (presenteeism) is scored as present if Yes on this question. For cost-effectiveness calculations, not conducted in this study, these can be expanded on by counting the hours and days concerned.
Job roles	iPCQ	Occupations as assessed by iCPQ^17^ were clustered according to International Standard Classification of Occupations (ISCO) classifications, as laid out by the International Labour Organisation (ILOSTAT).^53^	Job roles were organised into 7 categories: (a) management, (b) patient-facing, (c) research, (d) administrative, (e) elementary occupations, (f) patient-facing team lead/coordinator and (g) technical.	These distinctions were informed using ISCO groupings and were created due to the potential differences in working styles and responsibilities associated with these types of roles.

### Bias

In aiming to limit selection bias, we placed the study on the NIHR UK national research portfolio website,^36^ allowing any interested NHS trust to contact researchers directly to support recruitment (three NHS trusts were recruited using this method). The research team also approached the departments with academic staff of the participating university for participation, except those university departments with which the members of the research group were affiliated. Following receipt of informed consent, the EMPOWER app sent participants a series of reminders to complete baseline assessments. In addition, up to three email reminders (as appropriate) were sent from the research team to consenting individuals to encourage baseline assessment completion. Separately from the main study team, NHS research teams promoted recruitment and baseline assessment completion for the EMPOWER project by providing information to their staff members through organisational communication channels and posters.

### Sample size

The study planned to recruit at least 218 participants. This sample size is considered sufficient to enable a reliable analysis of up to 20 variables.^37,38^

### Statistical methods

Given the existing research literature suggesting relationships between mental health and presenteeism, a significance level of 0.05 was applied for all analyses. All analyses were performed in SPSS version 29.0.

### Descriptive statistics

To answer objective 1, descriptive statistics are provided for the total sample and separately for subgroups of participants with suicidal ideation and presenteeism, respectively, to ascertain the following: the types of work stressors frequently reported, the associated levels of work stress and psychological and somatic health and presenteeism. Patient Health Questionnaires PHQ-9^39^ and PHQ-8^40^ are both reported as measures of symptoms of depression within this research; PHQ-8 scores were used in analyses where suicidal ideation was included as a separate variable; analyses describing cut-off scores for severity of depression used PHQ-9.

### Subgroup analysis

Linear regressions (continuous variables) and chi-squared or Fisher’s exact estimates (categorical variables) were used to assess differences in responses between employees with and without suicidal ideation and presenteeism, respectively. Fisher’s exact estimates were used where the sample for analysis was insufficient to meet the assumption for Pearson chi-squared tests.^41^ Significance differences were indicated at *P = 0.05, **P = 0.01 and ***P = 0.001. Six Psychosocial Stressors at Work Scale (PSWS) items associated with higher stress scores were investigated to ascertain whether there were differences in the presence of these stressors for people in different job roles.

### Correlation analysis

To answer objective 2, we performed Spearman’s correlations to calculate associations in the full sample among work stress, suicidal ideation, symptoms of depression (PHQ-8) or anxiety, somatic symptoms, chronic medical conditions and presenteeism. When interpreting the strength of correlations, results between 0 and 0.39 were considered weak, between 0.40 and 0.69 were considered moderate and those between 0.70 and 1.00 were considered strong.^42^

### Sensitivity analysis

Given the focus on suicidal ideation in this study, we used PHQ-8 to assess depressive symptoms without taking suicidal ideation into account, and separately we used item 9 of PHQ-9 to assess suicidal ideation. In addition, to allow for comparison of symptoms of depression-related wider research literature in the field, we performed a sensitivity analysis by calculating correlations between symptoms of depression as measured by PHQ-9 and the other outcome variables, rather than by PHQ-8. To avoid multicollinearity, we did not perform this for suicidal ideation because that is assessed as part of PHQ-9. In addition, as a further sensitivity analysis, we explored whether there were significant differences between the university and the trusts in terms of work social context, job roles, suicidal ideation and presenteeism. Individual item scores from the Mini-Psychosocial Stressors at Work Scale (Mini-PSWS) were considered for the full sample and subsamples of participants (by organisation type, suicidal ideation and presenteeism) to ascertain the types of stressors frequently reported and the associated levels of stress. Chi-squared or Fisher’s exact estimates were used to assess differences in responses between participant groups.

### Missing data

Complete case data were used; individuals with incomplete responses for validated questionnaires were not included in analyses concerning those questionnaires. Numbers per questionnaire for the full sample are reported in Table 2.

**Table 2:** Descriptive characteristics for the full sample and subgroups with suicidal ideation and with presenteeism

Characteristics	Full sample	Subgroup no suicidal ideation	Subgroup suicidal ideation	Subgroup difference *P*-value	Subgroup no presenteeism	Subgroup presenteeism	Subgroup difference *P*-value
*N* (%)	328 (100)	291 (89)	37 (11)	–	144 (44)	183 (56)	–
Gender, *n* (%)	325 (99)						
Female	270 (82)	242 (83)	28 (76)		121 (84)	148 (81)	
Male	52 (16)	44 (15)	8 (22)	0.310	22 (15)	30 (16)	0.544
Other	3 (1)	3 (1)	0 (0)		0 (0)	3 (2)	
Age, *n* (%)	326 (99)						
Mean (s.d.)	42.82 (10.50)	43.25 (10.39)	39.51 (10.93)	**0.042**	43.20 (10.48)	42.45 (10.52)	0.522
Work stress, *n* (%)	325 (99)						
Mean (s.d.)	10.93 (10.16)	9.85 (8.91)	19.35 (14.60)	**<0.001**	7.73 (8.41)	13.45 (10.71)	**<0.001**
Depressive symptoms (PHQ-8), *n* (%)	328 (100)						
Mean (s.d.)	7.05 (5.18)	6.24 (4.58)	13.46 (5.19)	**<0.001**	5.17 (4.25)	8.57 (5.35)	**<0.001**
Suicidality (PHQ-9), *n* (%)	328 (100)						
No	291 (89)	291 (100)	0 (0)		**136 (94)**	**154 (84)**	**0.004**
Yes	37 (11)	0 (0)	37 (100)	–	**8 (6)**	**29 (16)**	
Depressive symptoms (PHQ-9), *n* (%)	328 (100)						
Mean total score (s.d.)	7.20 (5.40)	**6.24 (4.58)**	**14.73 (5.46)**	**<0.001**	**5.24 (4.38)**	**8.78 (5.61)**	**<0.001**
Depression threshold (PHQ-9)							
*n* (%) ≥5	201 (61)						
*n* (%) ≥10	98 (30)	**67 (23)**	**31 (84)**	**<0.001**	**23 (16)**	**75 (41)**	**<0.001**
Anxiety symptoms (GAD-7), *n* (%)	328 (100)						
Mean total score (s.d.)	5.87 (4.74)	**5.29 (4.36)**	**10.41 (5.22)**	**<0.001**	**4.60 (4.02)**	**6.87 (5.04)**	**<0.001**
Anxiety threshold							
*n* (%) ≥5	179 (55)						
*n* (%) ≥10	63 (19)	**44 (15)**	**19 (51)**	**<0.001**	**17 (12)**	**46 (25)**	**0.003**
Somatic symptoms (PHQ-15), *n* (%)	327 (100)						
Mean total score (s.d.)	7.85 (4.29)	**7.57 (4.17)**	**10.11 (4.58)**	**<0.001**	**6.39 (4.08)**	**9.01 (4.10)**	**<0.001**
Somatic symptom threshold							
*n* (%) ≥5	246 (75)						
*n* (%) ≥ 10	113 (34)	**91 (31)**	**22 (59)**	**<0.001**	**33 (23)**	**80 (44)**	**<0.001**
Medical conditions, *n* (%)	328 (100)						
No	194 (59)	176 (61)	18 (49)		92 (64)	102 (56)	0.142
Yes	134 (41)	115 (40)	19 (51)	0.214	52 (36)	81 (44)	
Presenteeism, *n* (%)	327 (100)						
No	144 (44)	**136 (47)**	**8 (22)**		144 (100)	0 (0)	
Yes	183 (56)	**154 (53)**	**29 (78)**	**0.004**	0 (0)	183 (100)	–

PHQ-8, Patient Health Questionnaire 8; PHQ-9, Patient Health Questionnaire 9; PHQ-15, Patient Health Questionnaire 15; GAD-7, Generalised Anxiety Disorder Screener.

Rounding in categorical variables may result in percentages that do not total 100 for some variables. Bold text represents significant results.

## Results

### Participants’ characteristics

**Fig. 1:**
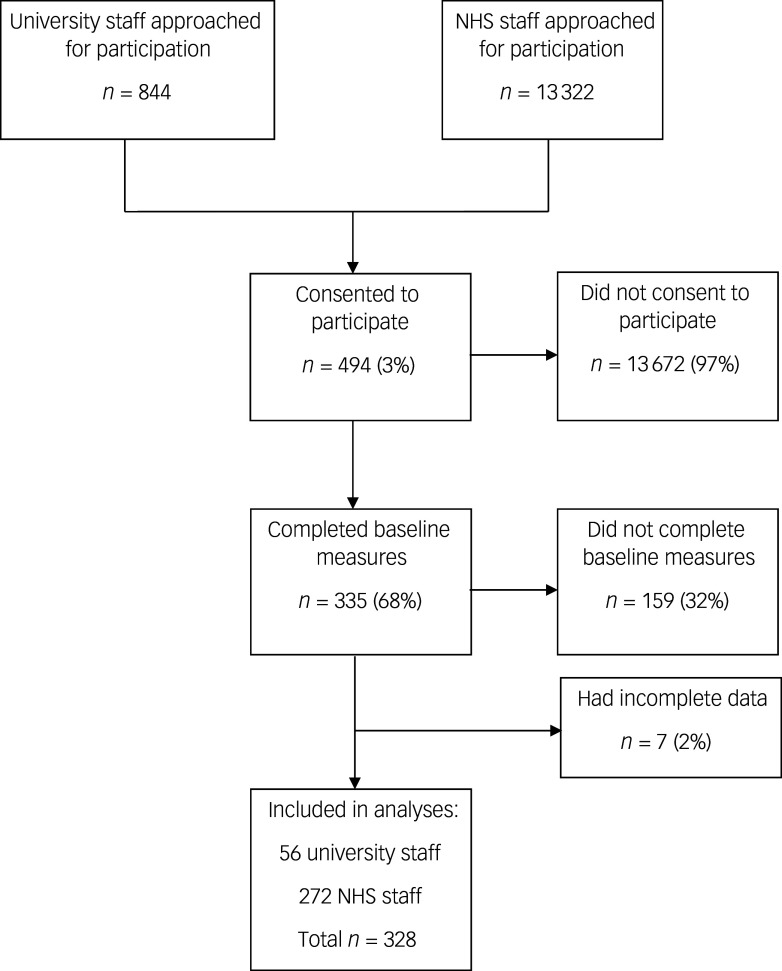
Flow chart of participants. NHS, National Health Service.

A flow chart of participants included in this study is shown in Fig. 1. Fifty-six university employees and 272 NHS staff were included.

### Descriptive data

Characteristics of the sample of 328 UK employees are shown in Table 2. The mean age of participants was 43 years; 82% were female.

### Main results

Two hundred and ninety-two participants (90%) scored some level of work stress (score >0), with a mean score of 10.93 (s.d. = 10.16). High levels of stress associated with work factors were found to relate to having too much work to do, tasks being perceived as too difficult, a lack of social support from supervisors or co-workers, having a bad working atmosphere, a poor work–home balance and working hours hindering private life (see Table 3). Thirty-seven employees (11%) reported suicidal ideation in the previous 2 weeks and 183 (56%) reported presenteeism. Mild or more serious depressive (61%), anxiety (55%) and somatic symptoms (75%) were reported. On average, those scores did not reach the highest severity cut-off point; however, in total, 30% scored above the highest threshold (≥10) for severe depressive, 19% for anxiety and 34% for somatic symptoms. A large proportion of respondents reported experiencing at least one chronic medical condition (*n* = 134, 41%).

**Table 3:** Frequency of stressors and associated level of work stress identified by subgroups with and without suicidal ideation and presenteeism

Stressors	Full sample *N* = 325		No suicidal ideation *n* = 288		Suicidal ideation *n* = 37		Subgroup difference *P*-value	No presenteeism *n* = 143		Presenteeism *n* = 182		Subgroup difference *P*-value
	Freq. (%)	LoS^1^ Mean (s.d.)	Freq. (%)	LoS^1^ Mean (s.d.)	Freq. (%)	LoS Mean (s.d.)		Freq. (%)	LoS Mean (s.d.)	Freq. (%)	LoS Mean (s.d.)	
Most tasks too difficult	10 (3)	3.50 (0.70)	**4 (1)**	**3.75 (0.50)**	**6 (16)**	**3.33 (0.82)**	**<0.001**	**1 (1)**	**2.00 (0)**	**9 (5)**	**3.67 (0.50)**	**0.047**
Most tasks too easy	40 (12)	1.48 (1.15)	33 (11)	1.33 (1.16)	7 (19)	2.14 (0.90)	0.284	20 (14)	1.25 (1.16)	20 (11)	1.70 (1.13)	0.497
Too much work to do	205 (63)	2.80 (0.91)	**175 (60)**	**2.69 (0.90)**	**30 (81)**	**3.43 (0.73)**	**0.018**	**75 (52)**	**2.53 (0.89)**	**130 (71)**	**2.95 (0.89)**	**<0.001**
Too little work to do	26 (8)	1.50 (1.14)	21 (7)	1.33 (1.11)	5 (14)	2.20 (1.10)	0.197	13 (9)	1.15 (0.90)	13 (7)	1.85 (1.28)	0.543
Working hours hinder private life	113 (35)	2.82 (0.97)	**94 (32)**	**2.72 (0.97)**	**19 (51)**	**3.30 (0.87)**	**0.028**	42 (29)	2.55 (0.99)	71 (39)	2.99 (0.93)	0.079
Limited influence on how their job was performed	80 (24)	2.65 (1.05)	**63 (22)**	**2.56 (1.06)**	**17 (46)**	**3.00 (0.94)**	**0.002**	**18 (13)**	**2.11 (0.96)**	**62 (34)**	**2.81 (1.02)**	**<0.001**
Lack of, or inappropriate, means to perform the job	74 (23)	2.71 (0.91)	62 (21)	2.60 (0.91)	12 (32)	3.25 (0.75)	0.147	29 (20)	2.60 (0.89)	45 (25)	2.78 (0.93)	0.355
Inappropriate work conditions	56 (17)	2.55 (1.03)	**45 (16)**	**2.42 (1.01)**	**11 (30)**	**3.09 (0.94)**	**0.039**	21 (15)	2.33 (0.97)	35 (19)	2.69 (1.05)	0.303
Poor communication within the organisation	122 (37)	2.67 (0.94)	103 (35)	2.63 (0.89)	19 (51)	2.89 (1.15)	0.073	**44 (31)**	**2.48 (0.93)**	**78 (43)**	**2.77 (0.93)**	**0.029**
Lack of social support from supervisors or co-workers	76 (23)	3.09 (0.85)	**62 (21)**	**3.00 (0.85)**	**14 (38)**	**3.50 (0.76)**	**0.038**	**25 (17)**	**2.84 (0.80)**	**51 (28)**	**3.22 (0.86)**	**0.034**
Bad working atmosphere	91 (28)	2.95 (1.03)	**75 (26)**	**2.85 (1.02)**	**16 (43)**	**3.37 (0.96)**	**0.033**	**29 (20)**	**2.76 (1.02)**	**62 (34)**	**3.03 (1.02)**	**0.006**
Unclear role in their team	76 (23)	2.71 (1.09)	**61 (21)**	**2.61 (1.10)**	**15 (41)**	**3.13 (0.99)**	**0.013**	**23 (16)**	**2.39 (1.16)**	**53 (29)**	**2.85 (1.05)**	**0.008**
Lack of opportunities for professional development	84 (26)	2.54 (1.25)	**68 (23)**	**2.43 (1.23)**	**16 (43)**	**3.00 (1.27)**	**0.016**	**25 (17)**	**2.28 (1.28)**	**59 (32)**	**2.64 (1.23)**	**0.003**
Need to adapt to continual changes	166 (51)	1.77 (1.18)	148 (51)	1.68 (1.15)	18 (49)	2.44 (1.20)	0.862	66 (46)	1.47 (1.03)	100 (55)	1.96 (1.23)	0.119
Poor work–home balance	105 (32)	2.86 (0.96)	88 (30)	2.78 (0.96)	17 (46)	3.24 (0.83)	0.064	40 (28)	2.50 (1.09)	65 (36)	3.08 (0.80)	0.153
Remote work	229 (70)	0.53 (0.93)	208 (72)	0.47 (0.86)	21 (57)	1.19 (1.37)	0.058	94 (65)	0.34 (0.74)	135 (74)	0.67 (1.03)	0.112

Freq., frequency; LoS, level of stress.

Bold text represents significant results. *P*-values are for tests run to compare the means.

### Subgroup analyses

Findings from two subgroups (employees with suicidal ideation and with presenteeism) are shown in Table 2. Table 3 shows the frequency of stressors, and the associated level of stress in both the total sample and subgroups with the presence of suicidal ideation and presenteeism.

Work stress levels were higher for those reporting suicidal ideation; the mean stress level for the majority (*n* = 15) of items in the PSWS scale was >2. The only exception to this was remote working, with a mean score of 1.19. Employees with suicidal ideation (11%) were found to be significantly younger, with worse scores for work stress, depressive, anxiety and somatic symptoms and presenteeism. A greater proportion had depressive, anxiety and somatic symptom scores reaching severe symptom levels than employees who did not report suicidal ideation.

Employees with presenteeism (56%) reported significantly worse scores for work stress, at levels higher compared with people without presenteeism for eight of the items in the PSWS scale. Items associated with mean scores >3 for the subgroup reporting presenteeism were: tasks being too difficult, lack of social support from supervisors or co-workers, a bad working atmosphere and having a poor work–home balance. In this subgroup, scores were also higher for suicidal ideation and depressive (PHQ-8), anxiety and somatic symptoms. A greater proportion had severe depressive, anxiety and somatic symptom level scores compared with employees without presenteeism.

### Correlations

Table 4 shows Spearman correlation analyses for outcome variables related to work stress. Most findings were significant. Work stress was found to be significantly associated with all factors except having chronic medical conditions, with effect sizes considered moderate. The two variables most strongly correlated were anxiety and depression symptoms (*r* = 0.679, *P* < 0.01).

**Table 4:** Spearman correlations

Outcome variables	Work stress	Depressive symptoms	Suicidal ideation	Anxiety symptoms	Somatic symptoms	Chronic medical conditions
Depressive symptoms	0.290**					
Suicidal ideation	0.225**	0.385**				
Anxiety symptoms	0.299**	0.679**	0.314**			
Somatic symptoms	0.245**	0.568**	0.172**	0.429**		
Chronic medical conditions	0.073	0.155**	0.076	0.097	0.271**	
Presenteeism	0.311**	0.334**	0.161**	0.241**	0.308**	0.082

**Correlation significant at the 0.01 level (2-tailed).

### Sensitivity analysis

Correlations between outcome measures and depressive symptoms (PHQ-9) are listed in Supplementary Table 2, and are in line with the correlations shown in Table 4.

### University and NHS trusts in terms of participants’ scores

There were no significant differences between the university and NHS trusts in terms of participants’ scores on PSWS (*P* = 0.319). There was no significant difference between NHS trusts and the university in terms of suicidal ideation and presenteeism, as shown in Supplementary Table 3. The most substantial difference between the university and NHS trusts in terms of job roles was the absence of patient-facing job roles in the university, whereas 64% of NHS responses were made by those in a patient-facing role such as a nurse, allied health professional, doctor or healthcare assistant (*P* < 0.001). Individuals working in research and administrative roles reported statistically significantly lower PSWS scores compared with those working in patient-facing roles, as presented in Supplementary Table 4.

### Missing data

The number of participants with incomplete baseline data (1 or more questionnaires missing) was minimal (*n* = 7); these participants were not included in the analysis. For those included, minimal missing data were noted for the following variables: gender (*n* = 3), age (*n* = 2), work stress total score (*n* = 3), somatic symptoms total score (*n* = 1) and presenteeism (*n* = 1).

## Discussion

### Main findings

This study found that 90% of the participants experienced some form of work stress. Over 50% of participants indicated experiencing workload pressures and a need to adapt to continual changes. These align with some of the previously identified drivers of presenteeism.^54^ The adverse work circumstances reported in this research appear to relate to task-related pressures, having no influence on role or career and unsatisfactory social networks within the team and wider organisation. The latter signals the importance of relationships among NHS staff at times of strain, such as the COVID-19 pandemic.^55–57^ The proportion of participants reporting these adverse work circumstances was typically higher in the subgroups who reported suicidal ideation and presenteeism. Studies during the earlier phase of the pandemic showed high work stress levels in university staff and students,^13^ and in NHS staff, in the UK;^56,57^ the current study shows that elevated work stress levels continue in the context of work in the later years of the pandemic. This suggests that individuals experiencing work stress may require additional support to address challenges in their workplaces. Managers could combine individual-level with organisational support. Policy-makers should consider structural or policy changes minimising psychosocial risks that would be optimal in reducing the effects of work stress on employees.

Suicidal ideation was reported in 11% of the total sample and 16% for employees reporting presenteeism. This is high compared with general population estimates: 3.96% in a systematic review of European general population studies conducted between 2008 and 2017.^58^ The UK Health and Safety Executive provides guidance for employers to reduce suicide risk among employees.^59^

Although suicide prevention initiatives have been developed and evaluated as effective at preventing completed suicides and attempted suicides in the general population and clinical settings,^60^ there appear to be limited initiatives specifically targeting employment settings. While the importance of providing support for mental health and prevent work-related suicide has been highlighted by the World Health Organization, opportunities to improve support for workers in this respect have not yet been optimised. Previous research has highlighted the negative impact of stigma on the willingness of people to engage with individual-level suicide prevention initiatives.^61^

Fifty-six per cent of the whole sample reported presenteeism, which is high compared with pre-pandemic estimates that suggested the prevalence rate of presenteeism to be approximately 40% over the previous year. The percentage is significantly higher (78%) in the group reporting suicidal ideation, suggesting that employees with presenteeism would benefit from a specific signposting approach to get help and treatment for suicidal ideation and associated mental disorders, and specific attention to alleviate work stressors. With a recent systematic review reporting that up to 70% of employees internationally showed sickness presenteeism and related mental health problems,^62^ this is an issue that needs to be addressed without delay. From these findings, there is an opportunity to develop stepwise interventions for people showing signs of presenteeism, with a first step being the provision of mental health awareness and support, and a second step to offer a suicide prevention intervention where this is appropriate. Research in this field is starting to explore ways to implement interventions of this type in the workplace, but further work in this area is required.

In this study we hypothesised that there would be an association between work stress, suicidal ideation, job roles, presenteeism, mental health conditions, somatic symptom burden and chronic medical conditions, and that these outcomes would be positively interrelated. The results of our analyses confirm these hypotheses to be true except for chronic medical conditions, where this is only partly the case. In this sample, a large proportion of participants reported at least one chronic medical condition (41%). This aligns with the high levels of chronic medical conditions previously reported in workforce research, and reinforces the need for employers to promote access to healthcare support for their employees. However, these conditions were not associated with work stress levels but rather with somatic and depressive symptoms. Furthermore, there were differences in the association between job roles and work stress, with employees in patient-facing roles experiencing significantly greater work stress than individuals working in research and administrative roles, which can be expected given the burden of contact with patients ill with COVID-19.

### Limitations

This study has several limitations, such as the cross-sectional nature of the design that does not allow exploration of causal relationships between variables, and the relatively small number of participants who completed baseline measures in relation to the number of individuals who consented to take part (68%). There was a large difference between the number of people approached to participate in this study and those who consented to participate (3%); this research was conducted in a stressful period during the COVID-19 pandemic, which may have influenced the number of people who agreed to participate. Given the voluntary nature of recruitment, explanations about who agreed to participate and why can only be speculative. However, despite measures taken to limit selection bias, this may well have occurred. The sample reported is a convenience sample that cannot be considered representative of all UK employees, nor indeed of all NHS or university employees in terms of demographic characteristics, because many standard demographic variables such as ethnicity were not collected. In addition, 82% of the participants were female. Although this reflects a relatively high percentage of female employees in both industries, the findings of this study cannot be generalised in terms of gender. It is a limitation that this study was conducted in only four NHS trusts – three of them mental health trusts and one for mental and physical health and disability – and one university, and did not include acute and intensive care employees, which means that generalisability of the findings is limited and replication in a larger sample would be advisable.

In addition, because the participants’ self-reported their responses to the items in the questionnaires, the symptoms of mental disorders reported do not represent psychiatric diagnoses. Mental health symptoms reported do not represent psychiatric diagnoses but only, in the case of scores indicating severe symptom levels, the possibility of the presence of mental health conditions as validated in the respective questionnaires,^39^ that would ‘require clinical evaluation to determine if the threshold for clinical action has been reached’.^63^ Nevertheless, scores on PHQ-9, Generalised Anxiety Disorder Screener (GAD-7) and PHQ-15 below severe symptom levels can still indicate high distress, which corroborates the fact that a large proportion of NHS staff were distressed during the pandemic. Regarding presenteeism, overall good content and construct validity and reliability of iPCQ for absenteeism has been demonstrated by validating the self-report against public registry data, which might be considered the ‘gold standard’. Testing the criterion validity of presenteeism, however, poses significant challenges due to the absence of a gold standard or objective measures.^29^ The self-reporting nature of the way in which data were collected to measure presenteeism in the iPCQ is therefore a limitation in the study reported here.

Although the proportion compared with previous research was high, only a relatively small number of people (*n* = 37) reported suicidal ideation, which resulted in unbalanced groups in suicidal ideation comparisons. This is a common phenomenon in suicidal ideation-related research. Also, although underreporting may have played a role here, this would apply to any research into suicidal ideation.

### Strengths

This is an innovative study exploring work stress and associated factors, including the association with both suicidal ideation and presenteeism. A strength of this work is the inclusion of assessments for somatic symptoms, suicidal ideation and presenteeism, which adds to the research literature on work stress and allows measurement of associations between these constructs. Our data show the extent of suicidal ideation in employees of four NHS trusts and one university in England, and suggest that there may be a relationship between suicidal ideation, work stress and presenteeism. This work underscores the importance of monitoring employees’ well-being. The findings from this work could therefore contribute to the development of policy guidelines to support employers in identifying and tackling work stress issues affecting their employees. In addition to considering work stress, suicidal ideation and presenteeism, this study included widely used validated measures of depressive, anxiety and somatic symptoms to support the assessment of mental health in employees. The findings of the sensitivity analyses indicated that the correlations between PHQ-9 were similar to those for PHQ-8, suggesting that our findings are robust.

### Recommendations for further research

Further research into factors influencing work stress, suicidal ideation, presenteeism and mental health as a component of the workplace is warranted. This could usefully include work context-related mechanisms leading to suicidal ideation in work stress, as well as secondary stressors such as the loss of colleagues due to COVID-19, and bereavement and distress such as the considerable length to which healthcare personnel would go to protect their families from contamination with COVID-19, as potential factors. These should also study how employees can be made aware of this and what interventions might be effective in improving mental well-being, including suicidal ideation. Researchers should now consider options for interventions supporting supervisors and managers in identifying employees at risk and signposting them towards help, to support them in fulfilling their legal responsibilities to protect their employees from consequences of work stress. Research aligning mental health support with interventions targeting presenteeism and suicidal ideation should also be explored.

### Policy recommendations

This study found high levels of work stress, suicidal ideation and presenteeism in employees in the settings of both a university and the NHS during the third and fourth years of the COVID-19 pandemic in the UK, and these are interrelated. These findings warrant the development of policies to address work stress and provide employee support in the work context. Given the impact of the COVID-19 pandemic on the workforce, there is a need for preventative interventions in the workplace to improve and support physical well-being and mental health. Where previous evidence for suicide prevention programmes exists, this should be extended to provide effective interventions within the workplace. These interventions should extend the learning gained from other settings to provide multi-level support and minimise potential stigma related to seeking help.

## Supporting information

van der Feltz-Cornelis et al. supplementary material van der Feltz-Cornelis et al. supplementary material

## Data Availability

The anonymous data reported in this paper can be made available to interested parties upon request to the lead author, as follows. Researchers can submit a research plan, which describes the background and methods of a proposed research question, and a request for specific data used in this study to answer the research question. Requests to access the data-set should be directed to christina.vanderfeltz-cornelis@york.ac.uk.

## Supplementary material

The supplementary material is available online at https://doi.org/10.1192/bjo.2025.10069
