# Supplementary material for: Work stress and its association with suicidal ideation, health and presenteeism during the COVID-19 pandemic: cross-sectional study in the UK health and university workforce
Source: BJPsych Open. 2025 Jul 21;11(4):e155. doi: 10.1192/bjo.2025.10069 (PMC12303827; doi:10.1192/bjo.2025.10069)
Supplement: van der Feltz-Cornelis et al. supplementary material [file S2056472425100690sup001.pdf]

Supplementary Materials

Supplementary Table 1: STROBE Statement—Checklist of items that should be included in reports of cross-sectional studies

	Item No	Recommendation	Page No
**Title and abstract**	1	(*a*) Indicate the study's design with a commonly used term in the title or the abstract	1
		(*b*) Provide in the abstract an informative and balanced summary of what was done and what was found	1
**Introduction**			
Background/ rationale	2	Explain the scientific background and rationale for the investigation being reported	2-4
Objectives	3	State specific objectives, including any prespecified hypotheses	4
**Methods**			
Study design	4	Present key elements of study design early in the paper	4
Setting	5	Describe the setting, locations, and relevant dates, including periods of recruitment, exposure, follow-up, and data collection	5
Participants	6	(*a*) Give the eligibility criteria, and the sources and methods of selection of participants. Describe methods of follow-up	5
		(*b*) For matched studies, give matching criteria and number of exposed and unexposed*	N/A
Variables	7	Clearly define all outcomes, exposures, predictors, potential confounders, and effect modifiers. Give diagnostic criteria, if applicable	Table 1
Data sources/ measurement	8	For each variable of interest, give sources of data and details of methods of assessment (measurement). Describe comparability of assessment methods if there is more than one group	Table 1
Bias	9	Describe any efforts to address potential sources of bias	5
Study size	10	Explain how the study size was arrived at	5
Quantitative variables	11	Explain how quantitative variables were handled in the analyses. If applicable, describe which groupings were chosen and why	Table 1
Statistical methods	12	(*a*) Describe all statistical methods, including those used to control for confounding	6
		(*b*) Describe any methods used to examine subgroups and interactions	6
		(*c*) Explain how missing data were addressed	7
		(*d*) If applicable, explain how loss to follow-up was addressed	N/A
		(*e*) Describe any sensitivity analyses	6
**Results**			
Participants	13	(a) Report numbers of individuals at each stage of study—eg numbers potentially eligible, examined for eligibility, confirmed eligible, included in the study, completing follow-up, and analysed	Fig. 1
		(b) Give reasons for non-participation at each stage	Fig. 1

		(c) Consider use of a flow diagram	Fig. 1
Descriptive data	14	(a) Give characteristics of study participants (eg demographic, clinical, social) and information on exposures and potential confounders<br>(b) Indicate number of participants with missing data for each variable of interest<br>(c) Summarise follow-up time (eg, average and total amount)	7<br>Table 2<br>7<br>Table 2<br>N/A
Outcome data	15	Report numbers of outcome events or summary measures over time	Table 2
Main results	16	(*a*) Give unadjusted estimates and, if applicable, confounder-adjusted estimates and their precision (eg, 95% confidence interval). Make clear which confounders were adjusted for and why they were included<br>(*b*) Report category boundaries when continuous variables were categorized<br>(*c*) If relevant, consider translating estimates of relative risk into absolute risk for a meaningful time period	7-8<br>Tables 3 & 4<br>N/A<br>N/A
Other analyses	17	Report other analyses done—eg analyses of subgroups and interactions, and sensitivity analyses	8-9<br>Sup. Table 2, 3, 4
**Discussion**			
Key results	18	Summarise key results with reference to study objectives	9-11
Limitations	19	Discuss limitations of the study, taking into account sources of potential bias or imprecision. Discuss both direction and magnitude of any potential bias	11
Interpretation	20	Give a cautious overall interpretation of results considering objectives, limitations, multiplicity of analyses, results from similar studies, and other relevant evidence	12
Generalisability	21	Discuss the generalisability (external validity) of the study results	12
**Other information**			
Funding	22	Give the source of funding and the role of the funders for the present study and, if applicable, for the original study on which the present article is based	13

COVID restrictions, consequences for NHS and vaccinations during the recruitment period.

At the recruitment period, between April 2022 and September 2023, lockdowns and other COVID related restrictions had been lifted as they took place between March 2020 and December 2021. https://www.instituteforgovernment.org.uk/sites/default/files/2022-12/timeline-coronavirus-lockdown-december-2021.pdf

However, during the recruitment period there was a high strain on mental healthcare as the number of referrals for specialist NHS mental health care reached a record high in England of 4.3 million referrals by the end of 2021. https://www.bbc.co.uk/news/health-60734769

And in April 2022, hospital trusts in England were under "enormous strain", with some so busy they were having to divert ambulances to other sites; 20 hospitals had issued such instructions in the past week. In October 2022, hospital waiting lists in England had reached seven million, their highest since records began. https://en.wikipedia.org/wiki/Timeline_of_the_COVID-19_pandemic_in_England_(2022)

NHS staff received first COVID vaccinations September 2021 and then rolled along with the Spring and Fall boosters in 2022 and thereafter. https://www.bbc.co.uk/news/health-58583610

Supplementary Table 2: Spearman's correlations for sensitivity analysis

	Workstress	Depressive symptoms	Anxiety symptoms	Somatic symptoms	Pain/ Discomfort	Chronic medical conditions
**Depressive symptoms**	.295**					
**Anxiety symptoms**	.299**	.680**				
**Somatic symptoms**	.245**	.564**	.429**			
**Pain/ Discomfort[a]**	0.089	.242**	.136*	.446**		
**Chronic medical conditions[a]**	0.073	.157**	0.097	.271**	.244**	
**Presenteeism**	.311**	.333**	.241**	.308**	.131*	0.082

* Correlation significant at the 0.05 level (2-tailed). ** Correlation is significant at the 0.01 level (2-tailed).

Supplemental Table 3. Job roles by organisation, suicidal ideation and presenteeism.

	Full Sample	NHS employees	University employees	Organisation P value	No Suicidal Ideation	Suicidal Ideation	Ideation P value	No Presenteeism	Presenteeism	Presenteeism P value
**N** (%)	328 (100)	272 (83)	56 (17)	-	291 (89)	37 (11)	-	144 (44)	183 (56)	-
**Role categories** (%) Management	39 (12)	24 (9)	15 (27)		36 (12)	3 (8)		18 (13)	21 (12)	
Patient facing	175 (53)	175 (64)	0 (0)		151 (52)	24 (65)		75 (52)	99 (54)	
Research	44 (13)	17 (6)	27 (48)		39 (13)	5 (14)		19 (13)	25 (14)	
Administrative	38 (12)	27 (10)	11 (20)	**<0.001[a]*** **	36 (12)	2 (5)	0.146[a]	18 (13)	20 (11)	0.982[a]
Elementary occupations	4 (1)	4 (2)	0 (0)		3 (1)	1 (3)		2 (1)	2 (1)	
Patient facing team lead	21 (6)	20 (7)	1 (2)		21 (7)	0 (0)		10 (7)	11 (6)	
Technical	7 (2)	5 (2)	2 (4)		5 (2)	2 (5)		2 (1)	5 (3)	

Bold text represents significant results.

Supplemental Table 4: Linear Regression of Work Stress total score accounting for Job Role

	Mean PSWS score (sd)	B	Sig.	95% Confidence Interval Lower Bound	Upper Bound
**Patient Facing (reference)**	12.08 (10.91)	12.08	**<.001**	10.59	13.57
Management	10.39 (7.84)	-1.69	0.346	-5.20	1.83
Research	6.68 (6.80)	-5.40	**0.001**	-8.71	-2.08
Administrative	8.50 (8.82)	-3.58	**0.046**	-7.10	-0.06
Elementary occupations	16.00 (20.79)	3.92	0.438	-6.01	13.85
Patient Facing Team Lead	12.33 (10.68)	0.25	0.913	-4.28	4.79
Technical	18.14 (8.88)	6.06	0.116	-1.51	13.63

Bold text represents significant results.

Psychosocial Stressors at Work Scale (33)

Below you can find the list of psychosocial features which are sometimes present in workplaces.
For each feature please indicate whether you think the feature is a characteristic of your workplace (yes or no) and how stressed that particular feature makes you feel (0 not at all to 4 very much).
If a feature is not a characteristic of your workplace, please mark "no" and move to next item.
Please mark your answers by marking X in the relevant box

	Is this feature present at your workplace		How stressed does this feature make you feel?				
	No	Yes	0 Not at all	1	2	3	4 Very much
1. Most of my work tasks are too difficult for me (e.g. too complex, unclear, above my qualifications)	☐	☐	☐	☐	☐	☐	☐
2. Most of my work tasks are too easy for me (e.g. too simply, below my qualifications)	☐	☐	☐	☐	☐	☐	☐
3. Too much work to do	☐	☐	☐	☐	☐	☐	☐
4. Too little work to do	☐	☐	☐	☐	☐	☐	☐
5. Working hours hindering private life	☐	☐	☐	☐	☐	☐	☐
6. Lack or limited influence on how the job is performed (e.g. timing, order or way of completing tasks)	☐	☐	☐	☐	☐	☐	☐
7. Lack or inappropriate means to perform the job (e.g. tools, equipment, consumables)	☐	☐	☐	☐	☐	☐	☐
8. Inappropriate work conditions (e.g. temperature, noise, dirt, lighting, discomfort, occupational hazards)	☐	☐	☐	☐	☐	☐	☐
9. Poor communication within the organisation (e.g. no access to information, partial, wrong or conflicting information)	☐	☐	☐	☐	☐	☐	☐
10. Lack of social support from supervisors or co-workers (e.g. in carrying out work, solving problems, resolving conflicts)	☐	☐	☐	☐	☐	☐	☐
11. Bad working atmosphere (e.g. conflicts, sense of insecurity, aggression, unfair treatment, low morale)	☐	☐	☐	☐	☐	☐	☐
12. Unclear role in a team (e.g. unclear responsibilities, conflicting demands, tasks going beyond formal work obligations)	☐	☐	☐	☐	☐	☐	☐
13. Lack of opportunity for professional development (e.g. to gain / develop skills and competences)	☐	☐	☐	☐	☐	☐	☐
14. The need to adapt to continual changes (e.g. legal, technological, knowledge development)	☐	☐	☐	☐	☐	☐	☐
15. Lack work-home balance	☐	☐	☐	☐	☐	☐	☐
16. Remote work	☐	☐	☐	☐	☐	☐	☐

© van der Feltz-Cornelis et al., 2023 (33)

van der Feltz-Cornelis CM, Shepherd J, Gevaert J, Van Aerden K, Vanroelen C, Cepa OB, Recio LG, Bernard RM, Vorstenbosch E, Cristóbal-Narváez P, Felez-Nobrega M, de Miquel C, Merecz-Kot D, Staszewska K, Sinokki M, Naumanen P, Roijen LH, van Krugten F, de Mul M, Haro JM, Olaya B. Design and development of a digital intervention for workplace stress and mental health (EMPOWER). Internet Interv. 2023 Nov 4;34:100689. doi: 10.1016/j.invent.2023.100689. PMID: 38054076; PMCID: PMC10694565.
